# Hypertriglyceridemia in Newly Diagnosed Acute Promyelocytic Leukemia

**DOI:** 10.3389/fonc.2020.577796

**Published:** 2020-11-25

**Authors:** Jianai Sun, Yinjun Lou, Jingjing Zhu, Huafei Shen, De Zhou, Lixia Zhu, Xiudi Yang, Mixue Xie, Li Li, Xianbo Huang, Mingyu Zhu, Yanlong Zheng, Wanzhuo Xie, Xiujin Ye, Jie Jin, Hong-Hu Zhu

**Affiliations:** ^1^ Department of Hematology, The First Affiliated Hospital of Medical School of Zhejiang University, Hangzhou, Zhejiang, China; ^2^ Zhejiang Province Key Laboratory of Hematology Oncology Diagnosis and Treatment, Hangzhou, China

**Keywords:** acute promyelocytic leukemia, hypertriglyceridemia, all-trans retinoic acid, body mass index, peroxisome proliferator-activated receptor gamma

## Abstract

The primary aim of the present retrospective study was to investigate lipid profiles and kinetics in acute promyelocytic leukemia (APL) patients. We analyzed 402 newly diagnosed APL patients and 201 non-APL patients with acute myeloid leukemia (as control). Incidence of hypertriglyceridemia in APL patients and non-APL patients was 55.82% and 28.4% (*p* = 0.0003). The initial levels of triglycerides, total cholesterol, high-density lipoprotein cholesterol and low-density lipoprotein cholesterol were higher in APL patients than in control (all *p* < 0.0001). In APL patients, triglyceride levels were significantly increased during induction treatment with all-trans retinoic acid and arsenic. Multivariable analysis showed that age, being overweight (body mass index ≥25) and APL were independent risk factors for hypertriglyceridemia in all patients before treatment. High triglyceride levels were not significantly associated with disease-free survival or overall survival in the APL patients. In summary, in the current study triglyceride levels were significantly elevated in APL patients before treatment, and they increased during induction treatment, but there were no significant corresponding effects on survival.

## Introduction

Dyslipidemia is reportedly often detected in cancer patients, and it has recently attracted increased attention due to its potential prognostic value of cancer and mortality from cardiovascular diseases (1–3). In recent years, progress has been made in the study of dyslipidemia in patients with hematological malignancies. In some studies, there have been lower levels of total cholesterol (TC), high-density lipoprotein cholesterol (HDL), and low-density lipoprotein cholesterol (LDL), and higher triglyceride (TG) levels in patients with hematological malignancies (4–6). Small sample sizes and a lack of subgroup analyses may be the cause of the inconsistent results. As early as 1997 Estey et al. (7) reported that the incidence of obesity in acute promyelocytic leukemia (APL) patients was significantly higher than that in other types of acute myelocytic leukemia (AML). Many subsequent studies also indicated that obesity may be associated with APL (8) and differentiation syndrome (9), and that it may be have substantial adverse effects on clinical outcomes (10–12). Mazzarella et al. (13) recently reported that obesity was a dependent risk factor for APL. However, the correlation between dyslipidemia and APL has not been reported. Therefore, a study investigating a homogeneous leukemia type with a uniform treatment protocol and an adequate sample size is required.

In this retrospective study, we aimed to conduct a comprehensive study of lipid metabolism in 402 APL patients and a uniform protocol with all-trans retinoic acid (ATRA) and arsenic combination treatment to assess dyslipidemia during treatment and a 1-year follow-up period.

## Materials And Methods

### Patients and Sample Collection

A total of 603 leukemia patients were included in this retrospective study, 402 APL patients and 201 non-APL acute myelocytic leukemia patients (non-APL patients). APL patients aged over 14 years who were admitted to the clinic center affiliated with the First Affiliated Hospital of Zhejiang University School of Medicine from January 2011 to December 2019 were included. For comparisons, we collected the data of AML patients without APL in our hospital from January 2017 to December 2017. The study was approved by the institutional Ethics Committee and it was conducted in accordance with the Declaration of Helsinki.

APL Patients were treated primarily in accordance with the Shanghai APL protocol (14). We, routinely, took orally ATRA at 25 mg/m^2^/day and were intravenous injected arsenic trioxide 10 mg/day, until complete remission was achieved. APL patients underwent 3 courses of consolidation therapy (homoharringtonine/Mitox/daunorubicin and cytarabine) and sequentially underwent a course of ATRA combined with arsenic trioxide, 14 days apart for 24 months. For the non-APL patients, 3 days of idarubicin (10 mg/m^2^/day) and 7 days of cytarabine (100 mg/m^2^/day) were performed as induction therapy. When complete remission was achieved, patients received several courses of conventional chemotherapy including treatment of cytarabine every 12 h for 6 days (1.5–3.0 g/m^2^), homoharringtonine for 3 days (2 mg/m^2^/day), cytarabine for 7 days (75 mg/m^2^ twice a day) and aclarubicin for 7 days (12 mg/m^2^/day); daunorubicin for 3 days (45 mg/m^2^/day) and cytarabine for 7 days (100 mg/m^2^/day); idarubicin for 7 days (6–8 mg/m^2^/day) and aclarubicin for 5 days (20 mg/m^2^/day).

Demographic and clinical data that were obtained from medical records included: age, sex, height, weight, body mass index (BMI), WBC, hemoglobin count, platelet counts, albumin, alanine transaminase, aspartate transaminase, serum creatinine, uric acid, lactate dehydrogenase, Sanz’s risk score (15), PML-RARa transcript isoform and additional cytogenetic aberrations. The concentration of total TG, TC, HDL, and LDL and serum glucose were measured in all patients before the initiation of chemotherapy, twice a week during the first 4 weeks of induction chemotherapy, before the second course of the treatment, and during the third, sixth, and twelfth months. Blood samples were collected and stored in tubes containing ethylene diamine tetraacetic acid, and plasma levels of fasting TC, TG, HDL, and LDL *via* enzymatic method (Boehringer Mannheim, Mannheim, Germany). The lipid abnormality status was determined according to the criteria described by the expert panel of the National Cholesterol Education Program Adult Treatment Panel III Third Report (16). The upper limits of normal TG, TC and LDL were 1.7 mmol/L (150 mg/dL), 6.1 mmol/L (234.6 mg/dL) and 4.0 mmol/L (152.9 mg/dL), respectively. The lower limit of normal HDL was 0.96 mmol/L (55.4 mg/dL). The reporting recommendations for tumor marker prognostic studies (REMARK) guidelines were used as reference (17). We collected a total of 25,783 samples.

### Definition of Variable

According to the 2019 ESC/EAS Guidelines, hypertriglyceridemia is defined as 1.7 mmol/L (150 mg/dL) (18). In TG-based analysis, the patients were categorized into two major groups: high triglyceride group (HTG group, TG ≥ 1.7 mmol/L) and non HTG group (TG < 1.7 mmol/L). Patients were categorized into underweight/normal (BMI < 25) and overweight (BMI ≥25) in accordance with the current World Health Organization criteria. The initial WBC counts of APL patients were evaluated and adjusted. WBC counts ≤10 × 10^9^/L were considered as low risk and > 10 × 10^9^/L were considered high risk (19). Disease-free survival (DFS) was only used in analyses of patients who achieved complete remission and it was measured from the date of achievement of remission until the date of relapse or death from any cause; at last follow-up, patients with unknown relapse or death were removed on the date of their last examination. Overall survival (OS) was applied to all patients and it was defined as the length of time from the date of diagnosis to the death from any cause; patients whose death was at last follow-up were removed on the last date that they were known to have been alive.

### Statistical Analysis

Data are presented as median and absolute range (non-normally distributed data) or frequencies. The Shapiro-Wilk test was used to assess the normality of data distributions. The Wilcoxon Mann-Whitney test was used to compare the distribution of numerical variables. The χ^2^ test was used in qualitative variables. The relationships between clinical factors and dyslipidemia were assessed using univariable and multivariable logistic regression models. All multivariable analyses were adjusted by sex and age. The Kaplan–Meier method was used to estimate univariate survival curves and the differences between curves were analyzed *via* the log-rank test. Multivariable Cox proportional hazard regression models were used to assess the prognostic impact of hypertriglyceridemia with regard to OS and DFS. Statistical analyses were performed using SPSS software, version 23.0, and *p* < 0.05 was considered statistically significant.

## Results

### Patient Characteristics

Our study included 402 APL patients and 201 non-APL patients. 22.1% (71/321) of the APL patients were overweight, and 16.4% (33/201) of the non-APL patients were overweight (chi square test, *p* = 0.1); the median BMI was 22.83 ± 3.32 kg/m^2^ in APL patients and 22.59 ± 3.08 kg/m^2^ in non-APL patients (Mann–Whitney test, *p* = 0.426). The initial concentration of TG, TC, HDL, and LDL in the APL patients were significantly higher than those in control (Mann–Whitney test, *p* < 0.001, < 0.001, = 0.002, < 0.001). The proportions of the APL and non-APL patients who had hypertriglyceridemia before treatment were 55.8% and 28.4%, respectively (chi square test, *p* < 0.001). Detailed characteristics of the study population are shown in **Table 1**.

**Table 1:** Characteristics of the Study Population According to the Type of Leukemia.

	Non-APL	APL	P-value
Age (years), range	48.59(18–83)	42.9 (14–84)	<0.001
Gender			0.21
Male, No. (%)	95(47.3)	212(52.7)	
Female, No. (%)	106(52.7)	190(47.3)	
Height (cm), range	164.59(146–185)	164.62(148–184)	0.968
Weight (kg), range	61.38(35.5–100)	62.16(38–101)	0.461
BMI(kg/m2), range	22.59(0.5–98.8)	22.83(0.2–117.2)	0.426
Underweight/normal,	168(83.6)	250(77.9)	0.1
BMI<25, No. (%)			
Overweight/obese,	33(16.4)	71(22.1)	
BMI≥25, No. (%)			
WBC(10 ×109/L), range	35.55(0.5–98.8)	12.70(0.2–117.2)	<0.001
≥10 ×109/L, No. (%) 97(48.3)		118(29.5)	<0.001
<10 ×109/L, No. (%) 104(51.7)		282(70.5)	
HBG*(g/L), range PLT*(10 ×109/L), range	90.96(48–161) 62.31(6–361)	88.59(41–157) 36.65(4–225)	0.325 <0.001
ALB*(g/L), range	40.47(28.5–53.4)	42.48(25.3–66.8)	0.095
ALT*(U/L), range	27.99(5–389)	32.68(4–560)	0.068
AST*(U/L), range	27.17(7–539)	31.49(6–367)	0.062
Cr*(μmol/L), range	63.74(32–170)	64.96(18–322)	0.315
UA*(μmol/L), range	270.21(96–578)	235.44(36–561)	0.288
LDH*(IU/L), range	708.38(129–1025)	465.49(115–4462)	<0.001
TG*(mmol/L), range	1.57(0.44–10.39)	2.29(0.43–20.86)	<0.001
HTG group	57(28.4)	211(55.8)	<0.001
TG≥1.70, No. (%)			
TC*(mmol/L), range	3.45±1.09(0.96–7.13)	4.28(1.96–7.68)	<0.001
HDL*(mmol/L), range	0.86(0.23–2.44)	0.97(0.15−4.27)	0.002
LDL*(mmol/L), range	1.84(0.11−3.67)	2.28(0.11–4.99)	<0.001
Glucose*(mmol/L), range	5.67(2.14–14.51)	6.65(3.35–33.71)	<0.001

APL, acute promyelocytic leukemia; non-APL, acute myeloid leukemia exclude of APL; BMI, body mass index; WBC, white blood cell; HBG, Hemoglobin count; PLT, platelet count; ALB, albumin; ALT, alanine transaminase; AST, aspartate transaminase; Cr, serum creatinine; UA, uric acid; LDH, lactate dehydrogenas; TG, triglyceride; HTG group, high triglyceride group ≥ 1.70 (mmol/L); TC, cholesterol; HDL, high-density lipoprotein cholesterol; LDL, low-density lipoprotein cholesterol.

### Relationships Between Hypertriglyceridemia and Clinical Factors

We used univariable and multivariable h to explore the correlation between the hypertriglyceridemia and other clinical factors. In univariable analysis BMI and leukemia type were both risk factors for hypertriglyceridemia (*p* < 0.001). The results indicated that being overweight (BMI ≥ 25) (odds ratio [OR] 1.160, 95% confidence interval [CI] 1.087–1.238, *p* < 0.001) and leukemia type (OR 3.558, 95% CI 2.312–5.477, *p* < 0.001) were associated with hypertriglyceridemia. In APL patients hypertriglyceridemia was significantly associated with age (OR 1.026, 95% CI 1.008–1.044, *p* = 0.004), being overweight (OR 1.149, 95% CI 1.053–1.254, *p* = 0.002), and higher WBC count (OR 1.022, 95% CI 1.005–1.040, *p* = 0.011). While PML-RARa transcript isoform (*p* = 0.185) and abnormal karyotype (*p* = 0.907) were not significantly associated with hypertriglyceridemia. Compared with non-APL patients, APL patients were younger (OR 0.971, 95% CI 0.954–0.988, *p* = 0.001), higher hypertriglyceridemia (OR 1.828, 95% CI 1.383–2.415, *p* < 0.001), and had lower white blood cells (OR 0.977, 95%CI 0.967–0.986, *p* < 0.001) and platelets (OR 0.981, 95% CI 0.975–0.987, *p* <0.001) before treatment. Detailed results of logistic regression modelsing are shown in **Table 2**.

**Table 2:** Logistic regression models evaluating the associations between clinical variables and hypertriglyceridemia in APL and non-APL patients.

All patients		Univariable analysis	P-value	Multivariable analysis	P-value
Dependent variable	Independent variable	OR (95% CI)		OR (95% CI)	
HTG	Age	1.009(0.998–1.020)	0.130	1.016(1.003–1.029)	0.019
	Gender	1.246(0.889–1.747)	0.202	1.143(0.767–1.705)	0.512
	BMI	1.168(1.097–1.242)	<0.001	1.160(1.087–1.238)	<0.001
	Leukemia type	3.185(2.154–4.710)	<0.001	3.558(2.312–5.477)	<0.001
Leukemia type	Age	1.025(1.013–1.038)	<0.001	1.030(1.012–1.048)	0.001
(non-APL)	Gender	1.259(0.872–1.816)	0.219	1.543(0.906–2.628)	0.110
	BMI	0.980(0.924–1.040)	0.507	0.994(0.910–1.086)	0.897
	TG	0.555(0.446–0.689)	<0.001	0.547(0.414–0.723)	<0.001
	WBC	1.020(1.013–1.027)	<0.001	1.024(1.014–1.034)	<0.001
	PLT	1.014(1.010–1.019)	<0.001	1.019(1.013–1.026)	<0.001
	LDH	1.001(1.000–1.001)	0.001	1.000(1.000–1.001)	0.161
**APL patients**		**Univariable analysis**	**P-value**	**Multivariable analysis**	**P-value**
HTG	Age	1.021(1.007–1.035)	0.002	1.026(1.008–1.044)	0.004
	Gender	1.564(1.039–2.354)	0.032	1.222(0.715–2.088)	0.463
	BMI	1.165(1.080–1.257)	<0.001	1.149(1.053–1.254)	0.002
	WBC	1.026(1.012–1.040)	<0.001	1.022(1.005–1.040)	0.011
	LDH	1.001(1.000–1.002)	0.001	1.001(1.000–1.001)	0.122
	transcript isoform				

APL, acute promyelocytic leukemia; non-APL, acute myeloid leukemia exclude of APL; HTG group, high triglyceride group ≥ 1.70 (mmol/L); BMI, body mass index; WBC, white blood cell; TG, triglyceride concentration; PLT, platelet count; LDH, lactate dehydrogenase; OR, odds ratios; CI, confidence interval; PML-RARα, promyelocytic leukemia-retinoic acid receptor alpha.

### Lipid Kinetics

**Figure 1:**
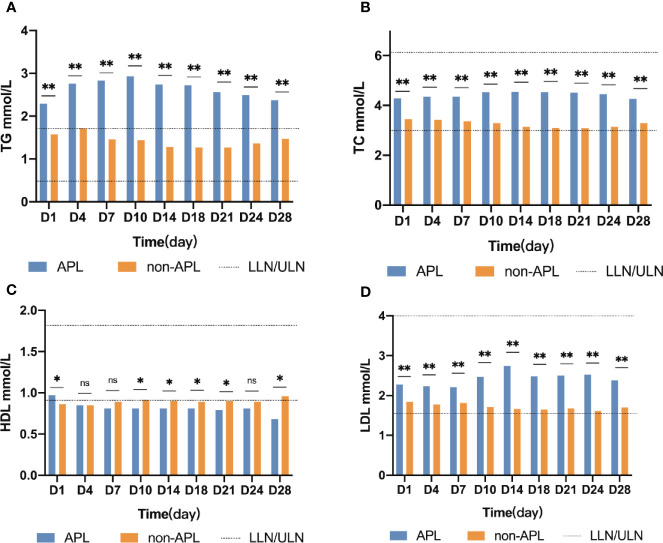
Lipid kinetics. Significant difference (*P < 0.05,**P < 0.0001); n.s., not significant. APL, acute promyelocytic leukemia; non-APL, acute myeloid leukemia exclude of APL; TG, triglyceride; TC, cholesterol; HDL, high-density lipoprotein cholesterol; LDH, low-density lipoprotein cholesterol; ULN, upper limits of normal; LLN, lower limits of normal.

Changes of TG levels during induction treatment are shown in **Figure 1**. TG concentrations were higher in APL patients than in non-APL patients at every timepoint investigated (*p* ≤ 0.001). The concentration of TG in APL patients continued to increase and peaked on day 10 (median 2.93, range 0.71–11.4, *p* < 0.001). In contrast, the concentration of TG in the non-APL patients followed a valley curve and reached the nadir on the day 18 (median 1.27, range 0.25–10.32, *p* < 0.001). Additionally, the TG concentration of the APL patients was higher than that of the non-APL patients at every timepoint (*p* ≤ 0.001). Furthermore, the median TG value in APL patients was higher than the upper limit of normal, and the corresponding value in the non-APL patients was lower than the upper limit of normal.

In non-APL patients TC, LDL, and serum glucose concentrations were significantly lower than those of APL patients, at all timepoints investigated (*p* < 0.05). However, the median TC, LDL, and glucose values in APL or non-APL patients at each time point were within the normal range. The detailed information is shown in **Figure 1**.

**Figure 2:**
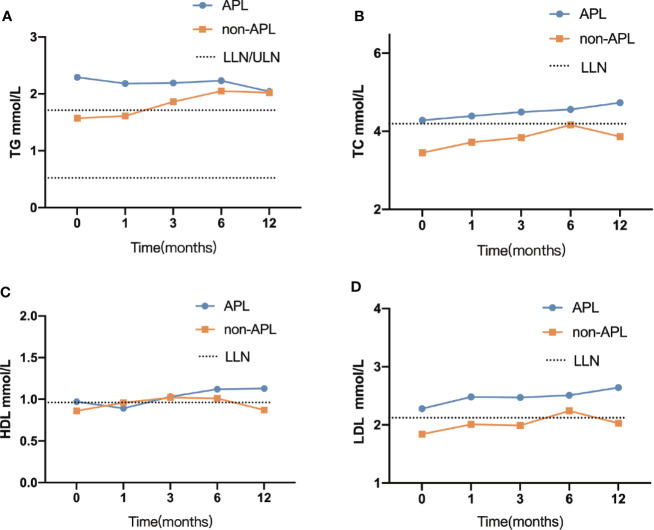
Comparison between the lipid profile at each time point. **(A)** TG, triglyceride **(B)** TC, cholesterol **(C)** HDL, high-density lipoprotein cholesterol **(D)** LDL, low-density lipoprotein cholesterol; APL, acute promyelocytic leukemia; non-APL, acute myeloid leukemia exclude of APL; ULN, upper limits of normal; LLN, lower limits of normal.

There was no significant difference in TG between APL and non-APL patients after 3 months, although the median was outside the normal range. At 12-month follow-up, there were 46.4% (83/179) of APL patients and 49.4% (88/178) of non-APL patients were hypertriglyceridemic. More detailed information pertaining to TG and hypertriglyceridemia is shown in **Figure 2**.

### Associations Between Hypertriglyceridemia and Survival in APL Patients

**Figure 3:**
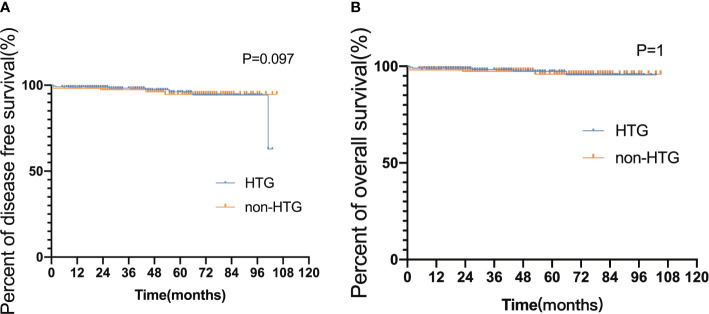
Disease-free survival and overall survival in HTG and non-HTG patients with APL. **(A)** Disease-free survival and **(B)** overall survival HTG group, high triglyceride group ≥ 1.70 (mmol/L); non-HTG group, triglyceride < 1.7 (mmol/L); Significant difference (P < 0.05).

The median follow-up time for the 353 surviving patients was 44 months (ranges 5–105 months). All APL patients experienced hematologic remission before maintenance therapy. 21 (early death rate 5.22%) patients died during induction therapy, 7 died after disease relapse, 3 survived after relapse, and 21 (5.2%) missed the follow-up. The 3-year DFS and OS rates were 92.65% and 93.12%, respectively. Neither DFS nor OS differed significantly in APL patients with and without hypertriglyceridemia (DFS hazard ratio [HR] 0.580, 95% CI 0.257–1.307, *p* = 0.097; OS HR 0.486, 95% CI 0.202–1.174, *p* = 1, **Figure 3**).

## Discussion

To the best of our knowledge the current study constitutes the first clinical evidence that TG levels are elevated in APL patients at the time of initial diagnosis compared with non-APL patients, and TG levels increased during induction therapy with ATRA and arsenic. Additionally, our results suggest that APL and being obesity (BMI ≧ 25) are risk factors for hypertriglyceridemia but hypertriglyceridemia is not significantly associated with DFS or OS in APL patients.

Chinese research (Chinese Chronic Diseases and Risk Factors Surveillance Research, n = 163,641, from 2013–2014) showed that the prevalence of hypertriglyceridemia was 25.8% in healthy adults which is higher than the 13.1% incidence reported in 2012 (20). In the USA, data from the National Health and Nutrition Examination Surveys showed that 47% of all adults in 2010 had hypertriglyceridemia (21). European data from 2016 show that 27% of all adults have a nonfasting triglyceride level >2.0mmol/l (22). Unsurprisingly, associations between hypertriglyceridemia and cancer have been reported in several studies (2, 3). Combined treatment with asparaginase and corticosteroids leads to hypertriglyceridemia in up to 67% of patients receiving treatment for acute lymphoblastic leukemia (5, 23). The hypertriglyceridemia incidence in APL patients is still unknown; our study found that the incidence of hypertriglyceridemia in Chinese APL patients before treatment was as high as 55.8%. After 1 year of follow-up, the incidence of hypertriglyceridemia in APL was still 46.4%.

We speculated that high TG levels—a risk factor for atherosclerotic cardiovascular disease—would lead to adverse prognoses. Large observational, epidemiological, genetic, and Mendelian randomization studies support the hypothesis that elevated blood triglyceride levels are independently associated with increased risk of atherosclerosis and coronary artery disease (22, 24, 25). TG levels > 1,000 mg/dL (11.4 mmol/L) can also induce acute pancreatitis (26). In the current study, only one APL patient had a lipid profile of 20.86 mmol/L. That patient did not develop acute pancreatitis during the course of treatment.

The mechanism of hypertriglyceridemia development in APL patients has not been completely clarified (27). We concluded that hypertriglyceridemia may be associated with APL as well as being overweight for three possible reasons. One pertains directly to being overweight, another pertains to the PML/RARα fusion protein, and the last involves the induction of abnormal lipid metabolism in APL patients by ATRA therapy.

With regard to being overweight, in a population-based cross-sectional study obesity was a significant risk factor for APL (13). The risk was particularly high in the APL subtype, with an estimated 44% HR increase per additional 5 kg/m^2^. Obesity is also associated with differentiation syndrome (9), relapse (10), and poor survival (11). Genetic up-regulation of pro-inflammatory factors related to direct growth promotion, generation of genotoxic oxidative stress, immune modulation (28, 29), and metabolic components of endogenous agonists for peroxisome proliferator-activated receptors (PPAR) (30, 31) may be involved in this clinical phenomenon in APL. While the pathophysiology of hypertriglyceridemia remains poorly understood (32), the mechanism of hypertriglyceridemia in the setting of obesity has been linked to insulin resistance. Not all obesity patients are insulin resistant (33), thus he interconnections involved need to be further explored.

With respect to the PML/RARα fusion protein, galectin-12 is selectively overexpressed in APL cells (34), and this overexpression is mediated by PPARγ (27). RAR activation leads to increased secretion and decreased catabolism of TG-rich particles, causing the accumulation of TG in the plasma and a secondary decrease in HDL-C levels. Because PML protein was initially described as a tumor suppressor, studies investigating PML have mainly focused on its roles in apoptosis, cell cycle regulation (35), tumorigenesis (36) and tumor metastasis (37). A growing number of studies have also observed an association between PML protein and metabolism. PML is upregulated in metastatic breast cancer and non-metastatic breast cancer with a poor prognosis, and studies indicate that PML may be involved in expression of the stem cell factor SOX9 and associated initiation of breast cancer (37). Carracedo and Pandolfi et al. (35) reported that hepatic PML protein levels are increased in obese individuals, suggesting that PML may be involved in hepatic function. Analyses of the microarray data in PML-depleted Human Umbilical Vein Endothelial Cells suggest that PML is involved in the regulation of a large number of metabolic genes (38, 39). The rearrangement of glucose and fatty acid metabolic gene expression in PML knockout mice, reportedly resulted in an increased metabolic rate and counteracted Western diet-induced obesity symptoms (40). In a previous study there was a negative correlation between PML-RARα expression and PPARγ in APL cells (41). Subsequent experiments suggest that the metabolic stress sensor Tribbles homolog 3 may inhibit the activity of PPARγ by interfering with interaction between PPARγ and RXR, and promoting the ubiquitination and degradation of PPARγ. The synergistic effect of PML-RARα and elevated Tribbles homolog 3 can inhibit the activity of PPAR and cause abnormal lipid metabolism in newly diagnosed APL patients. APL patients. Therefore, PML can be used as a nutritional sensor to maintain metabolic homeostasis.

Abnormal lipid metabolism in APL patients was induced by ATRA therapy. In APL patients ATRA reportedly stimulates the synthesis of cholesterol and triglycerides in the liver, elevating blood lipid levels (42). *In vitro* experiment indicated that TG levels in NB4 APL cell line treated with ATRA were significantly higher than TG levels prior to treatment (27). The G0/G1 switch gene 2 (G0S2) is also a direct PPARγ target gene and may be involved in the adipocyte differentiation (43). In another study adipose triglyceride lipase was a rate-limiting factor in the inhibition of TG metabolism by G0S2, which partially mediates the therapeutic effects of ATRA in APL by increasing TG levels (44). In addition, the transcription factor forkhead box O1 (FOXO1) is stimulated by retinoid therapy and is inactivated *via* phosphorylation of insulin. This inactivation leads to decreased gluconeogenesis and the release of very low-density lipoprotein by the liver after feeding. FOXO1 stimulates microsomal triglyceride transfer protein and apolipoprotein C-III, which lead to the activation of particle assembly and inhibition of lipoprotein lipase, the two —which are both causes of increased plasma TG (45). Interestingly, the retinoid effects on FOXO1-dependent increases in apolipoprotein C-III are inhibited by PPARγ (46).

Treatment for hypertriglyceridemia includes the management of lifestyle and secondary factors, and pharmacotherapy. The guidelines recommend that if the patient has conditions, such as type 2 diabetes, obesity, alcohol overuse, hypothyroidism, pregnancy, hepatosteatosis, renal failure, or concomitant drug use, the primary disease should be treated. The management of mild-to-moderate hypertriglyceridemia (< 10 mmol/L) should follow recommended guidelines with an initial emphasis on diet and exercise after these secondary conditions have been addressed. The potential benefits of using related inhibitors such as PPARγ agonizing thiazolidinediones require further investigation. Thiazolidinediones (rosiglitazone, pioglitazone) are oral insulin-sensitizing medications used in type 2 diabetes mellitus that can reduce glucose with a minimal risk of hypoglycemia and potential anti-atherosclerotic effects. Some studies suggested that thiazolidinediones can inhibit the proliferation of HL-60 cell lines (47). *In vitro*, pioglitazone induces chronic myelogenous leukemia cells to exit the quiescent state, thereby making them sensitive to the effects of imatinib. After promising results in a case series (48) and a single-arm phase 2 study (49), combination treatment with pioglitazone and imatinib is now under prospective randomized investigation (ClinicalTrials.gov Identifier NCT02767063). Whether it is necessary to use thiazolidinediones in combination in APL requires rigorous investigation.

As time went on, the blood lipid levels were similar in approximately 1 year. Gastrointestinal side effects after chemotherapy in non-APL patients may reduce their intake, but APL induction therapy has little effect on diet. The reason for this is unclear. After only one year of follow-up, the incidence of hypertriglyceridemia in APL was still 46.4%. This may be because they were still on retinoic acid maintenance treatment, or because many APL patients are prone to hypertriglyceridemia due to lifestyle habits such as excessive nutrient intake, low levels of exercise, and relatively low levels of participation in social activities. Our results suggested that elevated TG did not result in shorter DFS or OS under this protocol. Notably in this regard, the complications of associated with hypertriglyceridemia take a long time to manifest. Hypertriglyceridemia remains an important clinical phenomenon, and lipid metabolism is very important in APL.

There are several limitations to this study. The follow-up period was relatively short, limiting the conclusions that could be drawn about many common complications associated with hypertriglyceridemia such as arteriosclerosis and coronary heart disease. In addition, with the prolonged follow-up time, there are more data on blood lipid loss.

In summary, there are evidently significant associations between body weight and hypertriglyceridemia in APL patients, and potential relationships between them require further investigation. Hypertriglyceridemia may be related to the pathogenesis of APL and this may have implications with respect to treatment with ATRA. Relationships between APL, ATRA treatment, and lipid metabolism require further investigation, and such research may ultimately result in improved prognoses.

## Data Availability Statement

The raw data supporting the conclusions of this article will be made available by the authors, without undue reservation.

## Ethics Statement

The studies involving human participants were reviewed and approved by the Research Ethics Committee of the first affiliated Hospital, College of Medcine, Zhejiang University. Written informed consent from the participants’ legal guardian/next of kin was not required to participate in this study in accordance with the national legislation and the institutional requirements. Written informed consent was obtained from the individual(s) for the publication of any potentially identifiable images or data included in this article.

## Author Contributions

HZ contributed to the conception and design of the article. JS and YL wrote the manuscript. JJ revised the manuscript. All authors contributed to article revision, read, and approved the submitted version.

## Funding

This work was supported by grants from the National Natural Science Foundation of China (81970133 and 81820108004). The sponsor had no role in the design, analysis, interpretation, or publication of this study.

## Conflict of Interest

The authors declare that the research was conducted in the absence of any commercial or financial relationships that could be construed as a potential conflict of interest.

## References

[1] Kreger BE Anderson KM Schatzkin A Splansky GL Serum cholesterol level, body mass index, and the risk of colon cancer. The Framingham Study. Cancer (1992) 70(5):1038–43. 10.1002/1097-0142(19920901)70:5<1038::aid-cncr2820700505>3.0.co;2-m 1515981

[2] Fiorenza AM Branchi A Sommariva D Serum lipoprotein profile in patients with cancer. A comparison with non-cancer subjects. Int J Clin Lab Res (2000) 30(3):141–5. 10.1007/s005990070013 11196072

[3] Sun Y Meng H Jin Y Shi X Wu Y Fan D Serum lipid profile in gynecologic tumors: a retrospective clinical study of 1,550 patients. Eur J Gynaecol Oncol (2016) 37(3):348–52. 10.12892/ejgo2854.2016 27352562

[4] Scribano D Baroni S Pagano L Zuppi C Leone G Giardina B Return to normal values of lipid pattern after effective chemotherapy in acute lymphoblastic leukemia. Haematologica (1996) 81(4):343–45. 10.1016/S0268-9499(96)80021-X 8870380

[5] Halton JM Nazir DJ McQueen MJ Barr RD Blood lipid profiles in children with acute lymphoblastic leukemia. Cancer (1998) 83(2):379–84. 10.1002/(SICI)1097-0142(19980715)83:2<379::AID-CNCR24>3.0.CO;2-P 9669823

[6] Mulas MF Abete C Pulisci D Pani A Massidda B Dessi S Cholesterol esters as growth regulators of lymphocytic leukaemia cells. Cell Proliferation (2011) 44(4):360–71. 10.1111/j.1365-2184.2011.00758.x PMC6496738 21645151

[7] Estey E Thall P Kantarjian H Pierce S Kornblau S Keating M Association between increased body mass index and a diagnosis of acute promyelocytic leukemia in patients with acute myeloid leukemia. Leukemia (1997) 11(10):1661–4. 10.1038/sj.leu.2400783 9324286

[8] Tedesco J Qualtieri J Head D Savani BN Reddy N High Prevalence of Obesity in Acute Promyelocytic Leukemia (APL): Implications for Differentiating Agents in APL and Metabolic Syndrome. Ther Adv Hematol (2011) 2(3):141–5. 10.1177/2040620711408490 PMC3573402 23556085

[9] Jeddi R Ghedira H Mnif S Gouider E Fenaux P Meddeb B High body mass index is an independent predictor of differentiation syndrome in patients with acute promyelocytic leukemia. Leukemia Res (2010) 34(4):545–7. 10.1016/j.leukres.2009.09.017 19800119

[10] Breccia M Mazzarella L Bagnardi V Disalvatore D Loglisci G Cimino G Increased BMI correlates with higher risk of disease relapse and differentiation syndrome in patients with acute promyelocytic leukemia treated with the AIDA protocols. Blood (2012) 119(1):49–54. 10.1182/blood-2011-07-369595 22049518

[11] Castillo JJ Mulkey F Geyer S Kolitz JE Blum W Powell BL Relationship between obesity and clinical outcome in adults with acute myeloid leukemia: A pooled analysis from four CALGB (alliance) clinical trials. Am J Hematol (2016) 91(2):199–204. 10.1002/ajh.24230 26526191 PMC4724329

[12] Li S Chen L Jin W Ma X Ma Y Dong F Influence of body mass index on incidence and prognosis of acute myeloid leukemia and acute promyelocytic leukemia: A meta-analysis. Sci Rep (2017) 7(1):17998. 10.1038/s41598-017-18278-x 29269861 PMC5740068

[13] Mazzarella L Botteri E Matthews A Gatti E Di Salvatore D Bagnardi V Obesity is a risk factor for acute promyelocytic leukemia: evidence from population and cross-sectional studies and correlation with FLT3 mutations and polyunsaturated fatty acid metabolism. Haematologica (2020) 105(6):1559–66. 10.3324/haematol.2019.223925 PMC7271575 31515354

[14] Shen ZX Chinese guidelines for the diagnosis and treatment of acute promyelocytic leukemia. Zhonghua xue ye xue za zhi = Zhonghua xueyexue zazhi (2011) 32(12):885–6. 10.3760/cma.j.issn.0253-2727.2014.05.024 22339972

[15] Sanz MA Lo Coco F Martin G Avvisati G Rayon C Barbui T Definition of relapse risk and role of nonanthracycline drugs for consolidation in patients with acute promyelocytic leukemia: a joint study of the PETHEMA and GIMEMA cooperative groups. Blood (2000) 96(4):1247–53. 10.1016/S1246-7820(00)80033-6 10942364

[16] Expert Panelon Detection, Evaluation, and Treatment of High Blood Cholesterol in Adults. Executive Summary of The Third Report of The National Cholesterol Education Program (NCEP) Expert Panel on Detection, Evaluation, And Treatment of High Blood Cholesterol In Adults (Adult Treatment Panel III). Jama (2001) 285(19):2486–97. 10.1001/jama.285.19.2486 11368702

[17] McShane LM Altman DG Sauerbrei W Taube SE Gion M Clark GM REporting recommendations for tumour MARKer prognostic studies (REMARK). Br J Cancer (2005) 93(4):387–91. 10.1038/sj.bjc.6602678 PMC2361579 16106245

[18] Mach F Baigent C Catapano AL Koskinas KC Casula M Badimon L ESC/EAS Guidelines for the management of dyslipidaemias: lipid modification to reduce cardiovascular risk. Eur Heart J (2019) 00:178. 10.1093/eurheartj/ehz455 31504418

[19] Tallman MS Wang ES Altman JK Appelbaum FR Bhatt VR Bixby D Acute Myeloid Leukemia, Version 3.2019, NCCN Clinical Practice Guidelines in Oncology. J Natl Compr Cancer Netw JNCCN (2019) 17(6):721–49. 10.6004/jnccn.2019.0028 31200351

[20] Zhang M Deng Q Wang L Huang Z Zhou M Li Y Prevalence of dyslipidemia and achievement of low-density lipoprotein cholesterol targets in Chinese adults: A nationally representative survey of 163,641 adults. Int J Cardiol (2018) 260:196–203. 10.1016/j.ijcard.2017.12.069 29622441

[21] Carroll MD Kit BK Lacher DA Shero ST Mussolino ME Trends in lipids and lipoproteins in US adults, 1988-2010. Jama (2012) 308(15):1545–54. 10.1001/jama.2012.13260 23073951

[22] Nordestgaard BG Triglyceride-Rich Lipoproteins and Atherosclerotic Cardiovascular Disease: New Insights From Epidemiology, Genetics, and Biology. Circ Res (2016) 118(4):547–63. 10.1161/circresaha.115.306249 26892957

[23] Steinherz PG Transient, severe hyperlipidemia in patients with acute lymphoblastic leukemia treated with prednisone and asparaginase. Cancer (1994) 74(12):3234–9. 10.1002/1097-0142(19941215)74:12<3234::aid-cncr2820741224>3.0.co;2-1 7982187

[24] Sniderman AD Couture P Martin SS DeGraaf J Lawler PR Cromwell WC Hypertriglyceridemia and cardiovascular risk: a cautionary note about metabolic confounding. J Lipid Res (2018) 59(7):1266–75. 10.1194/jlr.R082271 PMC6027915 29769239

[25] Reiner Z Hypertriglyceridaemia and risk of coronary artery disease. Nat Rev Cardiol (2017) 14(7):401–11. 10.1038/nrcardio.2017.31 28300080

[26] Hegele RA Ginsberg HN Chapman MJ Nordestgaard BG Kuivenhoven JA Averna M The polygenic nature of hypertriglyceridaemia: implications for definition, diagnosis, and management. Lancet Diabetes Endocrinol (2014) 2(8):655–66. 10.1016/s2213-8587(13)70191-8 PMC4201123 24731657

[27] Gu W Hu S He B Qiu G Ma J Chen Z Metabolites of acute promyelocytic leukemia cells participate in contributing to hypertriglyceridemia induced by all-trans retinoic acid. Leukemia Res (2009) 33(4):592–4. 10.1016/j.leukres.2008.07.017 18722659

[28] Wang D Dubois RN Eicosanoids and cancer. Nat Rev Cancer (2010) 10(3):181–93. 10.1038/nrc2809 PMC2898136 20168319

[29] Trabanelli S Chevalier MF Martinez-Usatorre A Gomez-Cadena A Salomé B Lecciso M Tumour-derived PGD2 and NKp30-B7H6 engagement drives an immunosuppressive ILC2-MDSC axis. Nat Commun (2017) 8(1):593. 10.1038/s41467-017-00678-2 28928446 PMC5605498

[30] Kliewer SA Lenhard JM Willson TM Patel I Morris DC Lehmann JM A prostaglandin J2 metabolite binds peroxisome proliferator-activated receptor gamma and promotes adipocyte differentiation. Cell (1995) 83(5):813–9. 10.1016/0092-8674(95)90194-9 8521498

[31] Poloz Y Stambolic V Obesity and cancer, a case for insulin signaling. Cell Death Dis (2015) 6:e2037. 10.1038/cddis.2015.381 26720346 PMC4720912

[32] Kim DH Zhang T Ringquist S Dong HH Targeting FoxO1 for hypertriglyceridemia. Curr Drug Targets (2011) 12(9):1245–55. 10.2174/138945011796150262 21443465

[33] Eckel RH The complex metabolic mechanisms relating obesity to hypertriglyceridemia. Arterioscler Thromb Vasc Biol (2011) 31(9):1946–8. 10.1161/atvbaha.111.233049 21849700

[34] Xue H Yang RY Tai G Liu FT Galectin-12 inhibits granulocytic differentiation of human NB4 promyelocytic leukemia cells while promoting lipogenesis. J Leukocyte Biol (2016) 100(4):657–64. 10.1189/jlb.1HI0316-134R PMC6608023 27256573

[35] Carracedo A Rousseau D Douris N Fernández-Ruiz S Martín-Martín N Weiss D The promyelocytic leukemia protein is upregulated in conditions of obesity and liver steatosis. Int J Biol Sci (2015) 11(6):629–32. 10.7150/ijbs.11615 PMC4440252 25999785

[36] Bernardi R Pandolfi PP Role of PML and the PML-nuclear body in the control of programmed cell death. Oncogene (2003) 22(56):9048–57. 10.1038/sj.onc.1207106 14663483

[37] Martín-Martín N Piva M Urosevic J Aldaz P Sutherland JD Fernández-Ruiz S Stratification and therapeutic potential of PML in metastatic breast cancer. Nat Commun (2016) 7:12595. 10.1038/ncomms12595 27553708 PMC4999521

[38] Carracedo A Weiss D Leliaert AK Bhasin M de Boer VC Laurent G A metabolic prosurvival role for PML in breast cancer. J Clin Invest (2012) 122(9):3088–100. 10.1172/jci62129 PMC3433768 22886304

[39] Cheng X Kao HY Microarray analysis revealing common and distinct functions of promyelocytic leukemia protein (PML) and tumor necrosis factor alpha (TNFα) signaling in endothelial cells. BMC Genomics (2012) 13:453. 10.1186/1471-2164-13-453 22947142 PMC3542097

[40] Cheng X Guo S Liu Y Chu H Hakimi P Berger NA Ablation of promyelocytic leukemia protein (PML) re-patterns energy balance and protects mice from obesity induced by a Western diet. J Biol Chem (2013) 288(41):29746–59. 10.1074/jbc.M113.487595 PMC3795272 23986437

[41] Li K Wang F Yang ZN Cui B Li PP Li ZY PML-RARalpha interaction with TRIB3 impedes PPARgamma/RXR function and triggers dyslipidemia in acute promyelocytic leukemia. Theranostics (2020) 10(22):10326–40. 10.7150/thno.45924 PMC7481410 32929351

[42] Bonet ML Ribot J Palou A Lipid metabolism in mammalian tissues and its control by retinoic acid. Biochim Biophys Acta (2012) 1821(1):177–89. 10.1016/j.bbalip.2011.06.001 21669299

[43] Zandbergen F Mandard S Escher P Tan NS Patsouris D Jatkoe T The G0/G1 switch gene 2 is a novel PPAR target gene. Biochem J (2005) 392(Pt 2):313–24. 10.1042/bj20050636 PMC1316267 16086669

[44] Zagani R El-Assaad W Gamache I Teodoro JG Inhibition of adipose triglyceride lipase (ATGL) by the putative tumor suppressor G0S2 or a small molecule inhibitor attenuates the growth of cancer cells. Oncotarget (2015) 6(29):28282–95. 10.18632/oncotarget.5061 PMC4695060 26318046

[45] Sachini N Arampatzi P Klonizakis A Nikolaou C Makatounakis T Lam EW Promyelocytic leukemia protein (PML) controls breast cancer cell proliferation by modulating Forkhead transcription factors. Mol Oncol (2019) 13(6):1369–87. 10.1002/1878-0261.12486 PMC6547613 30927552

[46] Dowell P Otto TC Adi S Lane MD Convergence of peroxisome proliferator-activated receptor gamma and Foxo1 signaling pathways. J Biol Chem (2003) 278(46):45485–91. 10.1074/jbc.M309069200 12966085

[47] Hirase N Yanase T Mu Y Muta K Umemura T Takayanagi R Thiazolidinedione induces apoptosis and monocytic differentiation in the promyelocytic leukemia cell line HL60. Oncology (1999) 57(Suppl 2):17–26. 10.1159/000055271 10545799

[48] Prost S Relouzat F Spentchian M Ouzegdouh Y Saliba J Massonnet G Erosion of the chronic myeloid leukaemia stem cell pool by PPARγ agonists. Nature (2015) 525(7569):380–3. 10.1038/nature15248 26331539

[49] Rousselot P Prost S Guilhot J Roy L Etienne G Legros L Pioglitazone together with imatinib in chronic myeloid leukemia: A proof of concept study. Cancer (2017) 123(10):1791–9. 10.1002/cncr.30490 PMC5434901 28026860

